# Exploring the association between statins use or HMG-CoA reductase inhibition and migraine: a systematic review and meta-analysis

**DOI:** 10.1186/s10194-025-01957-w

**Published:** 2025-02-03

**Authors:** Hamdy A. Makhlouf, Amr K. Hassan, Nereen A. Almosilhy, Ahmed S.A. Osman, Shrouk Ramadan, Moaz Elsayed Abouelmagd

**Affiliations:** 1 Faculty of Medicine, Minia University, Minya, Egypt; 2 School of Medicine, University of California, Irvine, CA USA; 3 Medical Research Group of Egypt, Negida Academy, Arlington, MA USA; 4 Department of Pharmacology and Toxicology, Faculty of Pharmacy, Tanta University, Tanta, Egypt; 5 Faculty of Medicine, Ain Shams University, Cairo, Egypt; 6 Faculty of Medicine, Cairo University, Cairo, Egypt

**Keywords:** Migraine, Statins, HMG-CoA reductase, Monthly migraine frequency, Mendelian randomization study, Meta-analysis

## Abstract

**Background:**

Statins or 3‑hydroxy‑3‑methyl‑glutarylcoenzyme A (HMG‑CoA) reductase inhibitors are medications that act by reducing the cholesterol content of liver cells Moreover, statins have been found to improve endothelial function and reduce vascular wall inflammation. A growing body of research suggests that statins are associated with less risk of migraine, and they can be used to treat symptoms. However, the evidence has been inconclusive, so we aim to investigate the nature and strength of the effect of statins on the prevention and prophylaxis of migraines.

**Methods:**

We conducted a comprehensive systematic search across multiple electronic databases, including PubMed, Scopus, Web of Science, and the Cochrane Library, from inception until October 2024, to include studies on the association between statins use and migraine. The outcomes of interest involved the association of the HMG-CoA reductase gene with the risk of migraine, as well as the association and efficacy of statins in migraine patients.

**Results:**

Thirteen studies were included in our systematic review. Mendelian Randomization (MR) studies revealed that expression of HMGCR was associated with an increased risk of migraine with odds ratio (OR) ranging from 1.38 to 1.55 (*P* < 0.001). Three observational studies investigating the relationship between statins and migraine risk demonstrated a protective effect, with odds ratios ranging from 0.73 to 0.94 (*P* < 0.001). The findings suggest a significant reduction in overall migraine risk, particularly for migraines with aura and in patients with higher vitamin D levels. Meta-analysis of randomized controlled trials (RCTs) showed that statins significantly reduced monthly migraine frequency (MD= -3.16, 95%CI= [-5.79, -0.53]; *p* = 0.02, I2 = 79%; *P* = 0.03). RCTs supported the efficacy of statins in reducing migraine frequency, days, and intensity compared to placebo.

**Conclusions:**

Statins, already well-established for cardiovascular benefits, emerge as a promising dual-purpose therapy for many neurological disorders. The association between the HMGCR gene and increased migraine risk, coupled with the possible efficacy of statins in reducing migraine frequency, may open new avenues for migraine prophylaxis. However, the variability in study design hinders definitive conclusions, so larger studies with longer follow-ups are required to ascertain both findings.

**Supplementary Information:**

The online version contains supplementary material available at 10.1186/s10194-025-01957-w.

## Introduction

Migraines are severe primary headache disorders characterized by moderate to severe intensity pulsating headache that lasts between 4 and 72 h and is often accompanied by nausea, vomiting, and extreme sensitivity to light and sound [1, 2]. It is the sixth most common disorder in the world and one of the most common neurological disorders [3]. Migraine affects about 12% of the population and is more frequent in women more than men and young people more than old [4]. The main line of treatment for migraine includes an on-demand therapy for attacks, like non-steroidal anti-inflammatory drugs (NSAIDs), and a prophylactic treatment to prevent attacks. Prophylactic treatment for migraine is especially recommended for patients where migraine attacks have a profound impact on daily activities or quality of life, where acute medication is not sufficient when disability occurs on two or more days per month, or in the case of prolonged or frequent migraine auras [5]. Available prophylactic therapies for migraine include antidepressants, antihypertensives, anticonvulsants, calcium channel antagonists, pizotifen, and memantine, and recently, statins have been suggested as a prophylaxis [6].

Statins or 3‑hydroxy‑3‑methyl‑glutarylcoenzyme A (HMG‑CoA) reductase inhibitors are a class of drugs used mainly to lower cholesterol and prevent cardiovascular disease medications. They increase the expression of the low‑density lipoprotein (LDL) receptor, leading to the reduction of LDL serum levels [7]. Additionally, Statins can reduce very low-density lipoproteins (VLDL) by affecting the secretion of apolipoprotein B levels and have been found to improve endothelial function, enhance blood flow, and reduce vascular wall inflammation that could trigger migraines [8].

Statins or HMG-CoA reductase inhibitors have come to attention lately in the field of neurology. The use of statins was not only associated with a lower risk of Parkinson’s disease but has been investigated for possible use to treat Parkinson’s disease [9, 10]. Since migraines are often associated with changes in blood flow and neuroinflammation [11], targeting these pathways by statins may have therapeutic effects. Additionally, Chronic inflammation and oxidative stress are known contributors to migraine [12]. Therefore statins’ anti-inflammatory and antioxidative properties may assist in alleviating these conditions, potentially lowering the frequency or severity of migraines [13]. Buettner and Burstein found in 2015 that migraine attacks were reduced in patients on statins and vitamin D3 [14]. Moreover, recent randomized controlled trials (RCTs) suggested a beneficial effect of statins in migraine prophylaxis [15]. Furthermore, recent Mendelian Randomization (MR) studies on genes concerned with lipid metabolism, particularly the HMG-CoA reductase gene, were conducted to uncover any possible link between said genes and the development as well as the severity of migraine [16].

Considering these recent advances in the literature on the effectiveness of statins or HMG-CoA reductase inhibition on migraine prophylaxis, as well as their relationship to lower migraine risk, We conducted the first systematic review and meta-analysis to comprehensively assess the relationship of the HMG-CoA reductase gene with the risk of migraine and the efficacy of statins in migraine patients.

## Methods

### Protocol registration

Following the guidelines detailed in *The Cochrane Handbook* [17], we conducted this systematic review and meta-analysis and reported the results based on the Preferred Reporting Items for Systematic Reviews and Meta-Analyses (PRISMA) guidelines [18]. Our review’s predefined protocol is registered with the International Prospective Register of Systematic Reviews (PROSPERO) under the identifier CRD42024597789.

### Search strategy and data sources

We conducted a comprehensive systematic search across multiple electronic databases, including PubMed, Scopus, Web of Science, and the Cochrane Library, from inception until October 2024. The search strategy incorporated relevant keywords and Medical Subject Headings (MeSH) related to migraines associated with the HMG-CoA reductase (HMGCR) gene and statin medications, including simvastatin, atorvastatin, rosuvastatin, pravastatin, fluvastatin, and lovastatin. Specifically, our search strategy for PubMed was: (statin OR simvastatin OR atorvastatin OR rosuvastatin OR pravastatin OR fluvastatin OR lovastatin OR HMG-CoA OR HMGCR) AND (migraine), detailed search strategy and number of studies from each database are provided in supplementary Table 1. Duplicate records were identified and removed using EndNote software. Furthermore, we conducted a manual review of reference lists from relevant reviews to ensure comprehensive study selection.

### Selection criteria

Two independent reviewers initially screened the titles and abstracts of the retrieved studies to identify potentially relevant publications, using Rayyan software [19]. Full-text articles were then assessed against the inclusion criteria. Studies were included based on the following criteria: the population consisted of patients diagnosed with any type of migraine; the interventions or exposure involved statins (e.g., simvastatin, atorvastatin, rosuvastatin, pravastatin, fluvastatin, lovastatin) compared to any other treatment or placebo; the outcomes of interest involved the association of the HMG-CoA reductase gene or statins use with the risk of migraine, as well as the efficacy of statins in migraine patients. The study designs included RCTs and observational studies (including MR studies, cohort studies, cross-sectional studies, and case series). Exclusion criteria included secondary studies, editorials, conference abstracts, case reports, and studies written in languages other than English. A discussion was held in case of any disagreement, or a senior author was invited to reach a consensus.

### Data extraction

Data extraction was performed independently by the reviewers using a standardized Excel sheet, with a separate reviewer verifying the extracted information. Any disagreements were resolved through a re-review of the original publications and discussion. Data sources included original publications, supplementary materials, and information from ClinicalTrials.gov to ensure comprehensive and up-to-date findings. Extracted data included study identifiers (e.g., first author, publication year, trial design, and study locations), treatment duration, participant demographics (sample size, age, and gender), treatment regimens, key findings, and treatment efficacy and safety outcomes (Monthly Migraine Frequency (MMF), Monthly Migraine Days (MMD), and migraine intensity as well as total adverse events).

### Methodological quality assessment

The methodological quality of each included study was independently assessed by the reviewers. RCTs were evaluated using the Cochrane Risk of Bias tool (ROB-2), while observational studies including cohorts, cross-sectional, and case-control designs, were assessed with the Newcastle-Ottawa Quality Assessment Scale (NOS) [20, 21]. Any disagreements between reviewers were resolved through consensus or consulting the first author.

### Data synthesis and statistical analysis

Data analysis was conducted using RevMan software. Effect sizes for associations were represented as odds ratios (OR) and 95% confidence interval (95%CI), while mean differences (MD) were utilized to quantify effect sizes for MMD, MMF, and intensity. A random-effects model was employed for all analyses to account for the variability inherent in HMGCR inhibitors and control groups.

### Heterogeneity evaluation

Heterogeneity may exist due to differences in study designs, RCT methodology, and statistics between studies, so we performed heterogeneity analysis using Chi-Square test and the I² statistic. For I^2^ values < 25%,25- 50%, and greater than 50%, respectively, heterogeneity was considered low, moderate, or high [22, 23]. A p-value below 0.1 or I² above 50% was considered indicative of significant heterogeneity. In instances where significant heterogeneity was detected, a sensitivity analysis (leave-one-out approach) was conducted to determine the impact of excluding individual studies on the overall effect size [24].

## Results

### Literature search

**Fig. 1:**
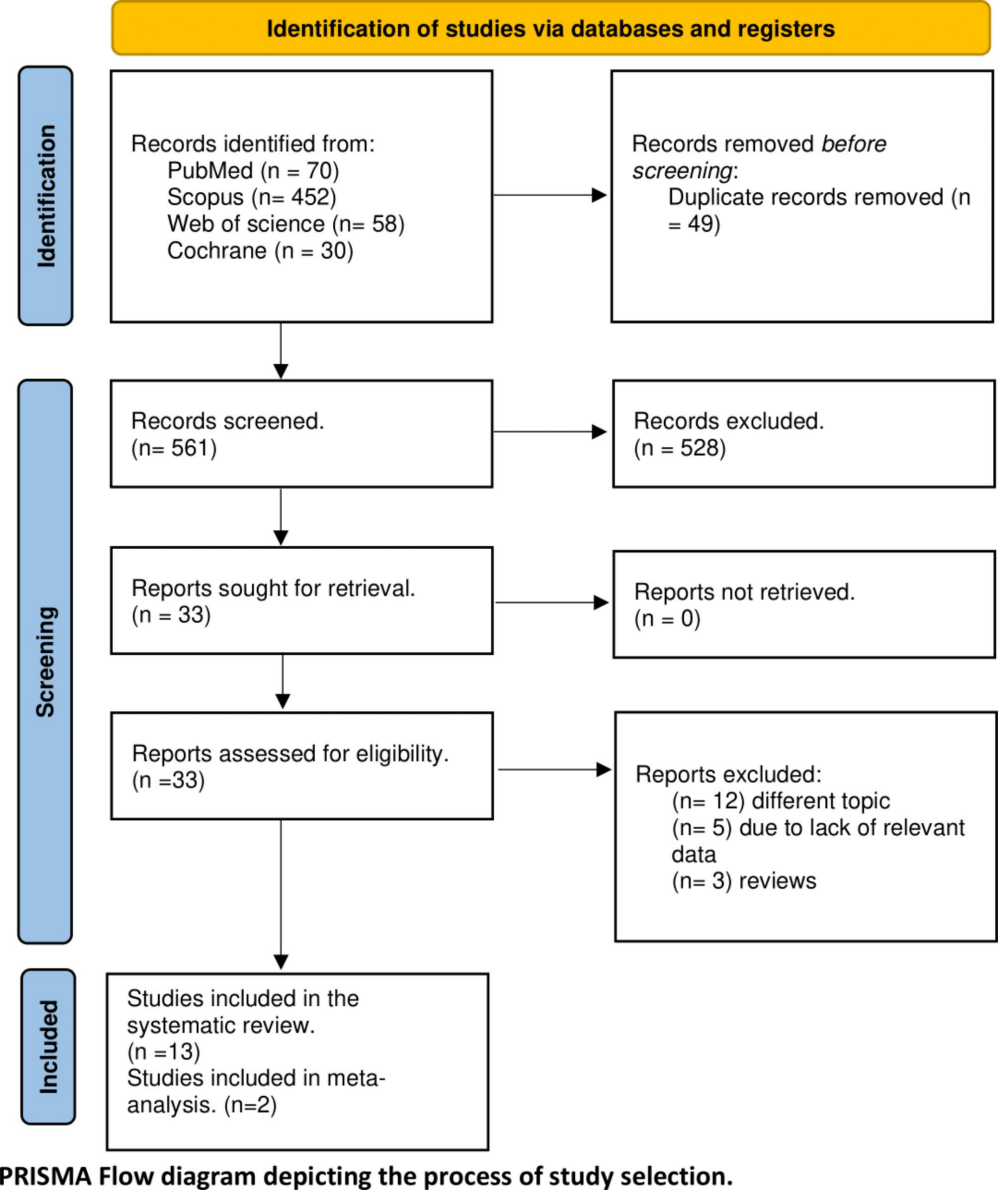
PRISMA Flow diagram of the process of study selection

Our literature search yielded 610 results, which became 561 after the exclusion of duplicates. Title and abstract screening resulted in 33 results, and after full-text screening, we included 13 studies in our review, involving 2 in the meta-analysis (Fig. 1).

### Characteristics of included studies

Our study included 13 studies. RCTs evaluated the efficacy of statins and combination therapies for migraine prevention, encompassing six studies from various countries with a total of 445 patients, most of them were female (86.5%). Most of our RCTs were conducted in Iran (4 studies) [25–28], while the other two were in Brazil [29] and the USA [15]. Treatment duration ranged from 4 weeks to 24 weeks. Detailed summary and baseline characteristics of the included studies highlighting the key findings of RCTs are shown in Tables 1 and 2. The rest of our included studies were seven observational studies varied in design, containing four MR studies [13, 16, 30, 31], two cohort studies [32, 33], and one cross-sectional study [14]. The Three MR studies addressed the association between the HMGCR gene and the risk of migraine, while others examined the association between statin use and migraine. Most studies were conducted in China (4 studies), with the rest from Norway, the USA, and Korea. Summary characteristics of observational studies and details about data sources are provided in Table 3.

**Table 1:** Summary characteristics of the included studies highlighting the key findings of RCTs

Study ID	Country	Study groups (intervention (Dose)/Control (Dose)	*N*	Inclusion criteria	Exclusion criteria	Treatment duration	Conclusion or key findings
**Buettner 2015** [15]	USA	**Intervention**: Simvastatin (20 mg twice daily) + Vitamin D3 (1000 IU twice daily) **Control**: Placebo	57	Adults ≥ 18 years with episodic migraine by ICHD-III criteria for ≥ 3 years and ≥ 4 migraine days/month	Chronic daily headache (≥ 15 headache days/month for ≥ 3 months), chronic opioid use, or conditions requiring or contraindicated to statins, severe renal disease, pregnancy, and nursing.	24 weeks	Simvastatin and vitamin D3 reduced migraine days by 8 in the first 12 weeks and 9 in the final 12 weeks (*p* < 0.001), while placebo increased days by 1 and 3, respectively (*p* < 0.001). By 24 weeks, 29% of the combination group achieved ≥ 50% reduction in migraine days versus 3% for placebo (*p* = 0.03). The combination also reduced abortive medication use and migraine disability with comparable adverse events, suggesting it as a potential preventive therapy.
**Ganji 2021** [25]	Iran	**Intervention**: Atorvastatin (20 mg) + Sodium Valproate (500 mg) **Control**: Placebo + Sodium Valproate (500 mg)	68	Patients aged 18–65, with a history of migraine with aura by ICHD-III criteria for at least 6 months, vitamin D3 > 30 ng/mL, and ≥ 3 migraine attacks monthly, but fewer than 3 high severity-migraine attacks with a negative impact on quality of life.	Chronic headaches (> 15/month), statin use for other conditions, liver/renal dysfunction, pregnancy.	8 weeks	Adding atorvastatin to sodium valproate reduced attack frequency (1.61 vs. 3.61, *p* = 0.0001) and pain severity (VAS: 3.27 vs. 5.87, *p* = 0.0001). Satisfaction was higher with atorvastatin (90.9% vs. 51.6%, *p* = 0.001). Mild side effects occurred in both groups, supporting atorvastatin as an effective, well-tolerated adjunct for migraine prophylaxis.
**Hesami 2018** [26]	Iran	**Intervention**: Atorvastatin (40 mg daily) **Control**: Sodium Valproate (500 mg daily)	82	Adults aged 18 to 50 years 6 to 15 migraine attacks per month in the last two months	Other types of headaches Current use of prophylactic treatment for migraine Pregnancy or breastfeeding Sensitivity to atorvastatin or sodium valproate Liver disease (Child-Pugh score B or C)	12 weeks	Atorvastatin and sodium valproate reduced migraine frequency, intensity, and duration with no significant differences between them. By three months, over 65% of patients in both groups achieved > 50% reduction in attack frequency (*P* = 0.499), and 70–75% had > 50% reduction in attack duration (*P* = 0.655). Analgesic use dropped by > 50% for over half of patients in both groups. Atorvastatin had fewer side effects (32% vs. 66%), suggesting it as an effective alternative for migraine prevention.
**Mazdeh 2020** [27]	Iran	**Intervention**: Propranolol (10 mg twice a day) + Rosuvastatin (10 mg daily) **Control**: Propranolol (10 mg twice a day) + Placebo	120	Age ≥ 18 years Migraine history of more than 3 years Migraine attacks ≥ 4 days/month Total cholesterol levels between 150–190 mg/dl	Pregnancy Breastfeeding Hepatic or renal failure Memory defects Sensitivity to statins Presence of atherosclerotic disorders	4 weeks	Propranolol and rosuvastatin reduced migraine attacks to 1.00 vs. 2.53 per week in controls (*p* < 0.001). Aura incidence was lower in the intervention group (1.7% vs. 6.7%, *p* = 0.171). Patients with sufficient vitamin D levels had fewer attacks (2.35 vs. 6.84, *p* = 0.002). No serious side effects were reported, supporting propranolol and rosuvastatin as effective migraine prevention with adequate vitamin D.
**Medeiros 2008** [29]	Brazil	**Intervention**: Simvastatin (20 mg) **Control**: Propranolol (60 mg).	54	women aged 18 to 45, (> 6/month) migraine attacks, additionally other group with hyperlipidemia	NA	90 days (about 13 weeks)	**Simvastatin and propranolol reduced migraine frequency in the last 30 days of treatment compared to baseline** (*P* < 0.05),** with consistent decreases observed each month throughout the trial.** Additionally, 88% of participants taking propranolol and 83% of those taking simvastatin experienced over a 50% reduction in migraine frequency, however, the difference in responder rates was not statistically significant (*P* = 0.7112), **with no significant difference in adverse effects.**
**Sherafat 2022** [28]	Iran	**Intervention**: Atorvastatin 40 mg + Nortriptyline 25 mg **Control**: Placebo + Nortriptyline 25 mg	68	Aged 18–65 years, with migraine based on the IHS criteria, (> 4/month) migraine attacks, (> 2/month) severe migraine attacks affecting quality of life, Vitamin D3 serum level > 30 ng/ml, no mood disorders, and basic educational attainment to respond to questionnaires.	History of liver disease, severe renal failure (GFR < 30 mL/min), atorvastatin use for other conditions, atorvastatin allergy, chronic headaches (15 + attacks/month), recent herbal or magnesium use, pregnancy or lactation, and low medication adherence (MMAS-8 score < 4).	24 weeks	By week 24, atorvastatin combined with nortriptyline reduced migraine attacks by 46% compared to nortriptyline alone (OR = 0.54, *P* = 0.007). Fewer than one attack per month was reported by 85.5% of atorvastatin patients vs. 47% in the control group (*P* = 0.004). Quality-of-life scores improved significantly (18.97 vs. 17.47), with no difference in headache intensity. Statins may effectively prevent migraines with mild side effects.

USA (United states of America), ICHD-III (International Classification of Headache Disorders, 3rd edition), IU (International Units), SD (Standard Deviation), VAS (Visual Analog Scale), OR (Odds Ratio), GFR (Glomerular Filtration Rate), MMAS-8 (Morisky Medication Adherence Scale-8), QOL (Quality of Life), NA (Not Applicable)

**Table 2:** Baseline characteristics of RCTs including clinical data of migraine (frequency or days)

Study ID	Intervention	Sample size	Age (Years) Mean (SD)	Gender (Female) *N*. (%)	Migraine frequency Mean (SD)	Total adverse events (%)
**Buettner 2015** [15]	Simvastatin + Vitamin D3	28	36.3 (18)	27 (96%)	MMD = 24.67 (15.23)	2 (7.1%)
	Placebo	29	28 (10.13)	25 (86%)	MMD = 18.3 (7.01)	6 (20.7%)
**Ganji 2021** [25]	Atorvastatin + Sodium Valproate	33	39.06 (26.34)	22 (66.6%)	MMF = 4.67 (1.05)	10 (30.3%)
	Placebo + Sodium Valproate	31	36.8 (23.31)	21 (67.7%)	MMF = 4.61 (1.09)	6 (19.4%)
**Hesami 2018** [26]	Atorvastatin	46	33.56 (8.51)	45 (97.8%)	MMF = 10.37 (3.25)	15 (32.6%)
	Sodium Valproate	36	33.25 (9.91)	34 (94.4%)	MMF = 11.14 (2.45)	24 (66.6%)
**Mazdeh 2020** [27]	Propranolol (10 mg twice a day) + Rosuvastatin (10 mg daily)	60	33.72 (10.16)	56 (93.3%)	NA	NA
	Propranolol (10 mg twice a day) + Placebo	60	36.27 (10.58)	56 (93.3%)	NA	NA
**Medeiros 2008** [29]	Simvastatin	25	NA	25 (100%)	MHD = 26 (3)	3 (9.37%)
	Propranolol	29	NA	29 (100%)	MHD = 19 (3)	3 (10.7%)
**Sherafat 2022** [28]	Atorvastatin 40 mg + Nortriptyline 25 mg	34	28.44 (7.84)	23 (69.7%)	> 3 attacks/month in 21 (61.76%) of patients	NA
	Placebo + Nortriptyline 25 mg	34	30.76 (5.78)	22 (64.7%)	> 3 attacks/month in 28 (82.6%) of patients.	NA

MMD (Monthly Migraine Days), MMF (Monthly Migraine Frequency), MHD (Monthly Headache Days), NA (Not Available)

**Table 3:** The characteristics of the included observational studies with details about data sources, migraine number, and key findings

Study ID	Study design	country	Source of data	Study duration	Migraine number	Control number	Variables/ genes addressed	Follow-up	Conclusion or key findings	NOS
**Bi 2023** [30]	Mendelian randomization study	China	FinnGen dataset and Choquet dataset (using data from the Genetic Epidemiology Research in Adult Health and Aging (GERA) cohort and UK Biobank)	NA	FinnGen(8547),Choquet (28,852)	FinnGen(176107),Choquet (525,717)	HMGCR	NA	Genetic proxies for HMGCR inhibition were significantly linked to a lower risk of migraine in the FinnGen dataset (OR = 0.64, 95% CI: 0.46–0.88, *p* = 0.0006) and near-significant in the Choquet dataset (OR = 0.78, 95% CI: 0.60–1.01, *p* = 0.06). Combined analysis confirmed a reduced migraine risk overall (OR = 0.73, 95% CI: 0.60–0.89, *p* = 0.0016). LPL enhancement genetic mimicry was also associated with decreased migraine risk in both FinnGen (OR = 0.82, 95% CI: 0.69–0.96, *p* = 0.01) and Choquet datasets (OR = 0.91, 95% CI: 0.83–0.99, *p* = 0.03). **Among the 10 lipid-lowering drug targets investigated**,** LPL and HMGCR showed significant associations with migraine risk. These findings indicate that LPL and HMGCR have the potential to serve as candidate drug targets for the treatment or prevention of migraines.**	6/6
**Hong 2024** [16]	Mendelian randomization study	China	The data source for the study was derived from a GWAS that was correlated with serum lipid levels, using genetic instrumental variables selected based on Graham et al. (2021). The dataset included a subset of individuals of European ancestry.The migraine-correlated GWAS summary data set was obtained from the IEU.	NA	8547	176,107	HMGCR	NA	Specific genetic variants related to LDL-C levels showed distinct effects: HMGCR variants were linked to an increased risk of migraines (OR = 1.46, *p* = 0.035) and MA (OR = 2.03, *p* = 0.008), PCSK9 variants were associated with a decreased risk of migraines (OR = 0.75, *p* = 0.001) and MA (OR = 0.69, *p* = 0.004), and APOB variants correlated with a reduced risk of MO (OR = 0.62, *p* = 0.000). **These results suggest that lipid metabolism characteristics are related to the risk of developing migraines.**	6/6
**Qu 2024** [13]	Mendelian randomization study	China	Summary-level GWAS data for migraines were obtained from a meta-analysis conducted by the International Headache Genetics Consortium (IHGC) and the FinnGen Consortium.	NA	GWAS = 48,975, FinnGen = 15,905	GWAS = 540,381, FinnGen = 264,662	quantitative trait loci of the HMG-CoA reductase gene and genetic variation within or near the HMG-CoA reductase gene region.	NA	High expression of HMG-CoA reductase was associated with an increased risk of migraines (OR = 1.55, 95% CI 1.30–1.84, *P* = 6.87 × 10⁻⁷) and confirmed by FinnGen (OR = 1.38, 95% CI 1.14–1.67, *P* = 7.38 × 10⁻⁴). Similarly, three genetically determined HMG-CoA reductase-mediated lipids were associated with an increased risk of migraine LDL-C (OR = 1.51, 95% CI 1.21–1.88, *P* = 2.50 × 10⁻⁴), TC (OR = 1.63, 95% CI 1.30–2.06, *P* = 2.93 × 10⁻⁵), and APOB (OR = 2.12, 95% CI 1.56–2.87, *P* = 1.35 × 10⁻⁶). In FinnGen, the APOB association was replicated (OR = 1.63, 95% CI 1.15–2.29, *P* = 5.60 × 10⁻⁴), while LDL-C (OR = 1.19, 95% CI 0.91–1.56, *P* = 0.20) and TC levels (OR = 1.21, 95% CI 0.95–1.53, *P* = 0.12) showed no significance. **These results support the potential use of HMGCR inhibitors in reducing migraine risk.**	6/6
**Zhang 2024** [31]	Mendelian randomization study	China	GWAS, brain eQTL data from the PsychENCODE consortia (*n* = 1,387), and cis-eQTLs for 16,987 genes derived from (31,684 blood samples) collected from healthy individuals	NA	GWAS = 48,975	GWAS = 540,381	HMGCR	NA	21 migraine-associated druggable genes were identified, with HMGCR and TGFB3 showing significance in both blood and brain tissues. HMGCR was linked to LDL levels, and TGFB3 to IGF1 levels. **These findings provide promising leads for more effective migraine treatments**,** potentially reducing drug development costs.**	6/6
**Study ID**	**Study design**	**country**	**Source of data**	**Study duration**	**Migraine number**	**Control number**	**Statin type in details**	**Follow-up**	**Conclusion or key findings**	**NOS**
**Bjørk 2023** [32]	Nationwide registry- based cohort study	Norway	Norwegian Prescription Database	10 years (2010–2019)	104,072 (Simvastatin was prescribed to 14,298 of them)	Other prescribed treatments	Simvastatin	at least one year - from 2010 to 2019	Compared to beta-blockers, botulinum toxin (HR 0.43, 95% CI 0.42–0.44), CGRP antibodies (HR 0.63, 95% CI 0.59–0.66), simvastatin (HR 0.71, 95% CI 0.69– 0.72), and candesartan (HR 0.76,95% CI 0.75– 0.77) had the lowest discontinuation rates, while clonidine (HR 2.95, 95% CI 2.88–3.02), topiramate (HR 1.34, 95% CI 1.31–1.37) and amitriptyline (HR 1.03, 95% CI 1.02– 1.05) were most frequently discontinued. Simvastatin, CGRPabs, and amitriptyline were more effective in reducing triptan use within 90 days, with ORs of 1.28 (95% CI 1.19– 1.38), 1.23 (95% CI 0.79– 1.90), and 1.13 (95% CI 1.08– 1.17), respectively. **Overall**,** CGRPabs**,** amitriptyline**,** and simvastatin showed better outcomes than beta blockers**,** while clonidine and topiramate showed poorer results.**	8/8
**Buettner 2014** [14]	Cross-sectional population-based study	USA	data from the National Health and Nutrition Examination Survey (NHANES)	3 Years (2001–2004)	1069	4869	NA	NA	Statin use was associated with a reduced prevalence of severe headache or migraine (OR: 0.67; 95% CI: 0.46–0.98; *p* = 0.04). A significant interaction was observed between statin use and serum vitamin D levels (*p* = 0.005). Among individuals with 25(OH)D > 57 nmol/l, statin use significantly lowered the odds of severe headache or migraine (OR: 0.48; 95% CI: 0.32–0.71; *p* = 0.001). However, no significant association was found for those with 25(OH)D ≤ 57 nmol/l. **These findings suggest that statin use**,** in combination with higher vitamin D levels**,** is associated with a reduced risk of severe headaches or migraines.**	**6/7**
**Kang 2024** [33]	Nationwide Korean Cohort	Korea	the Korean National Health Insurance Service-Health	from between 2002 and 2019	38,957	155.828	simvastatin, atorvastatin, pravastatin, lovastatin, rosuvastatin, and fluvastatin.	2 years	Statin use was associated with a reduced likelihood of migraines overall OR = 0.93 (95% CI = 0.91–0.95, *p* < 0.001), particularly for migraines with aura OR = 0.75 (95% CI = 0.65–0.86, *p* < 0.001) and without aura OR = 0.94 (95% CI = 0.92–0.96, *p* < 0.001). **Lipophilic statins were effective for both subtypes**,** while hydrophilic statins primarily reduced migraines without aura. Benefits were consistent across demographics but varied based on weight**,** smoking**,** alcohol use**,** anemia**,** and dyslipidemia history. Thus**,** statin therapy showed potential for migraine prevention**,** requiring tailored treatment based on patient profiles.**	8/8

GWAS (Genome-Wide Association Study), OR (Odds Ratio), CI (Confidence Interval), LDL-C (Low-Density Lipoprotein Cholesterol), TC (Total Cholesterol), APOB (Apolipoprotein B), TGFB3 (Transforming Growth Factor Beta 3), IGF1 (Insulin-Like Growth Factor 1), HR (Hazard Ratio), NHANES (National Health and Nutrition Examination Survey), CGRP (Calcitonin Gene-Related Peptide), 25(OH)D (25-Hydroxyvitamin D)

### Risk of bias assessment

**Fig. 2:**
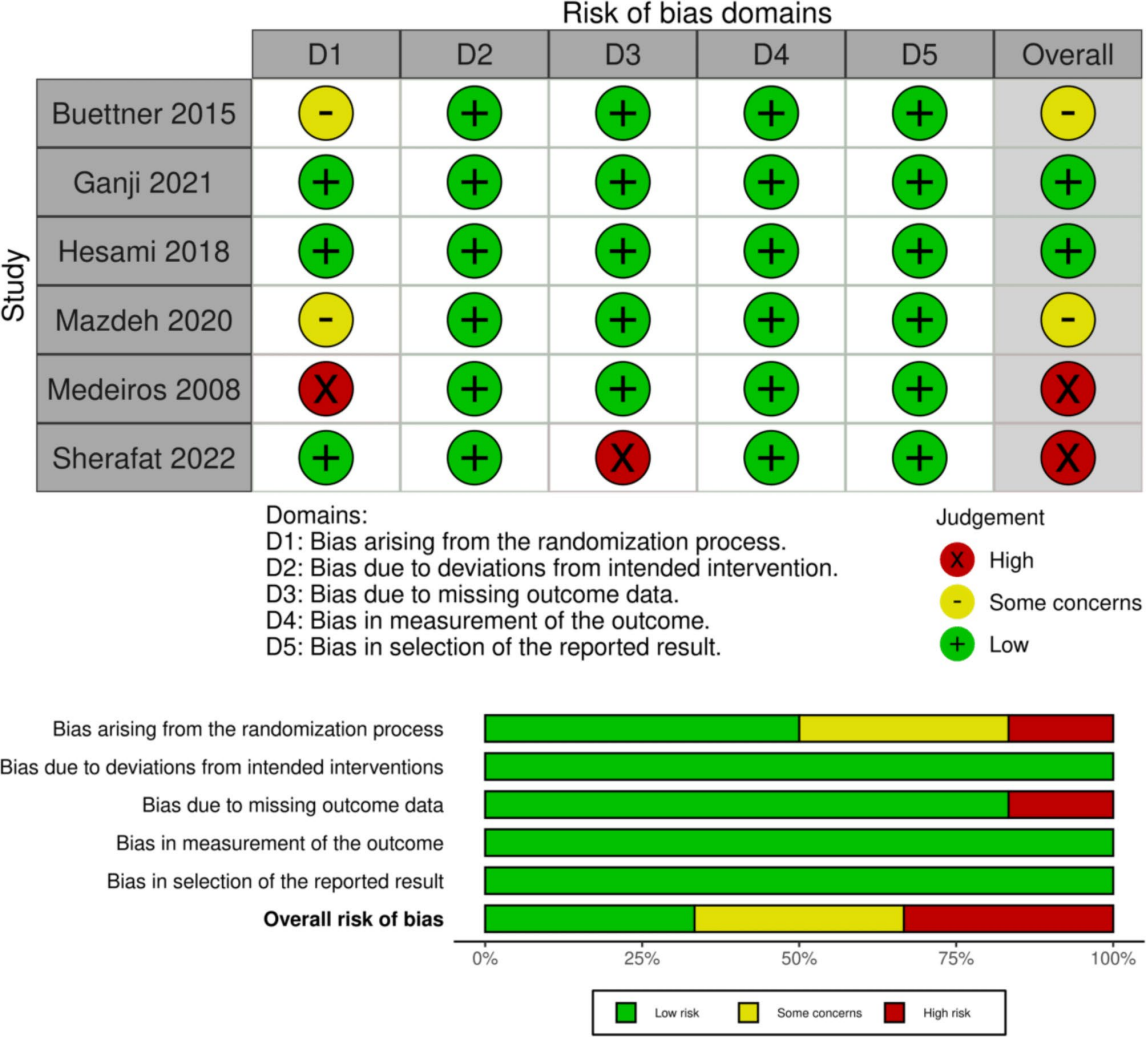
Results of risk of bias assessment by RoB2

Two of the included RCTs had a low risk of bias and followed the standards closely [25, 26]. However, another two trials revealed a high risk of bias due to errors in the randomization method and missing outcome data [28, 29]. The latter two studies raised some concerns about possible biases in the randomization process [15, 27], for further information see Fig. 2. We applied NOS to observational studies; all studies were found to be of high quality except Buettner 2014 was of moderate quality due to self-reported data [14]. Score of each study is shown in Table 3.

### Analysis of observational studies

#### Association of HMGCR gene activation with risk of migraine

Studies that examined the association between the HMGCR genes and migraine have all involved overlapping datasets in their analyses. Given the risk of multiple pooling of the same group of patients multiple times, no meta-analysis could be provided for this outcome. Hong et al. found that activation of HMGCR genes was associated with an increased risk of migraine (OR = 1.46, 95% CI= [1.03, 2.07]; *P* = 0.035), migraine with aura (OR = 2.03, 95% CI = [1.2, 3.42]; *P* = 0.008), but not migraine without aura (OR = 1.04, 95% CI = [0.6, 1.81]; *P* = 0.876) [16]. Qu et al. used data from the International Headache Genetics Consortium (IHGC) and revealed that expression of HMGCR was associated with an increased risk of migraines (OR = 1.55, 95%CI= [1.30, 1.84]; *P* < 0.001). They corroborated their findings by doing an analysis of the IHGC and FinnGen datasets, showing similar results (OR = 1.38, 95%CI= [1.14, 1.67]; *P* < 0.001) [13]. Finally, Zhang et al. found that HMGCR was significantly associated with the high risk of migraine in both blood (OR = 1.38, 95%CI= [1.21, 1.57]; *P* < 0.001) and brain (OR = 2.02, 95%CI= [1.49, 2.74]; *P* < 0.001) [31].

#### Association of statins or HMGCR inhibition with risk of migraine

Three studies have explored the relationship between statins or HMGCR inhibition and the risk of migraine. However, due to their different study designs, we could not conduct a meta-analysis on them. In 2014, Buettner et al. conducted a cross-sectional study examining the relationship between statin use with vitamin D status and severe headaches or migraine [14]. Their analysis of 5938 US participants demonstrated that statin use was significantly associated with a lower prevalence of severe headache or migraine (OR = 0.67, 95% CI= [0.46, 0.98]; *P* = 0.04), especially in patients who had serum 25-hydroxy vitamin D > 57 nmol/l (OR = 0.48, 95% CI= [0.32, 0.71]; *P* < 0.001) [14]. The MR study by Bi et al. supported these findings by revealing that HMGCR inhibition had a significant association with a lower incidence of migraine in the FinnGen dataset (OR = 0.64, 95% CI= [0.46, 0.88]; *P* < 0.001) and amarginal non-signficant association in the Choquet dataset (OR = 0.78, 95% CI= [0.60, 1.01]; *P* = 0.06) [30]. A combined analysis of these datasets showed a reduced overall migraine risk (OR = 0.73, 95% CI= [0.60, 0.89]; *P* < 0.001) [30]. Recently, a 2024 Nationwide Korean Cohort by Kang et al. further confirmed that statin use was associated with a reduced likelihood of overall migraines (OR = 0.93, 95% CI= [0.91, 0.95]; *P* < 0.001), particularly for migraines with aura (OR = 0.75, 95% CI = [0.65, 0.86]; *P* < 0.001) and without aura (OR = 0.94, 95% CI = [0.92, 0.96]; *P* < 0.001) [33].

#### Effect of statin on triptan use for migraine

According to A nationwide registry-based cohort study by Bjørk 2023, Out of 6096 Statin-using patients, 56.71% had a 30% decrease in triptan consumption over the first ninety days of therapy, with propensity score-adjusted odds ratios of 1.28, 95%CI= [1.19, 1.38) [32].

### Analysis of clinical trials

#### Statins efficacy on migraine frequency and days

**Fig. 3:**
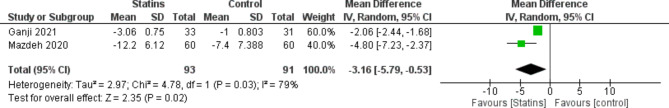
Meta-analysis of statins efficacy on monthly migraine frequency

Two RCTs reported MMF with 93 statin users and 91 controls. The findings demonstrated that statins were significantly associated with reduced MMF versus placebo (MD= -3.16, 95%CI= [-5.79, -0.53]; *p* = 0.02), albeit with substantial heterogeneity (*P* = 0.03; I2 = 79%) due to the variability of treatment regimens in each study (Fig. 3). This aligns with the results reported by Sherafat in 2022 which indicated that the combination of atorvastatin and nortriptyline reduced the risk of headache attacks by 46% when compared to nortriptyline alone (OR = 0.54; 95%CI= [0.34, 0.85]; *P* = 0.007) [28]. Additionally, Buettner et al. revealed that simvastatin plus vitamin D significantly reduced MMD compared to placebo in adults with episodic migraines (MD = -11.33, 95%CI= [-14.20, -8.46]; *P* < 0.001) [15].

#### Statins compared to other drugs

In 2008, Medeiros et al. compared Simvastatin 20 mg to Propranolol 60 mg and found that both simvastatin and propranolol significantly reduced MMD in women with Simvastatin showing greater decrease (MD = -20.65, 95%CI= [-21.06, -20.24], *P* < 0.001) than propranolol (MD = -14.85, 95%CI= [-15.44, -14.26], *P* < 0.001). In addition to reducing the frequency of migraine attacks, 88% of participants taking propranolol and 83% of those taking simvastatin experienced over a 50% reduction in migraine frequency [29]. Lately, in 2018, Hesami et al. randomized 46 patients to Atorvastatin (40 mg daily) and 36 patients to Sodium Valproate (500 mg daily) and found the numbers of patients with more than 50% reduction in the number of attacks (responder rate) were 30 (65.2%) in atorvastatin group and 26 (72.2%) in sodium valproate (*P* = 0.499), and both treatments reduced intensity, and duration of migraine attacks with less adverse events observed in Atorvastatin group, suggesting that atorvastatin could be a good alternative for migraine prophylaxis [26].

#### Statins safety and tolerability for migraine

The included studies demonstrated that statins maintained a favorable safety profile, showing results comparable to those of the control groups. At the same time, the event rate of all adverse events for statins was markedly less than sodium valproate (32% vs. 66%). However, the heterogeneous nature of the arms of the studies included in the meta-analysis precluded the conclusion of any significant adverse events to compare between the statin group and other groups. We also provided a summary of all adverse events in Table 2.

## Discussion

Our systematic review included 13 studies. Overall, our review indicated that statins had a significant association with a lower incidence of migraine particularly for migraines with aura or especially in patients who had serum 25-hydroxy vitamin D > 57 nmol/l. Additionally, statins were associated with decreased migraine frequency and triptan usage among migraine patients. RCTs included in our analysis suggested that statins might offer benefits similar to standard prophylactic treatments with a good safety profile, supporting their potential as alternative migraine prophylactics.

The direct evidence on the effect of HMGCR inhibition and risk of migraine was derived from 2 observational studies and one MR study. Although Buettner et al. may be limited by depending on self-reported data, the nested-case-control study by Kang et al. provides better evidence. Kang et al., not only the only study including other than the Western population (Koreans) but also shed light on the effect of migraine type and statin use. They found a larger effect of statin use on decreasing the risk of migraines with aura (OR = 0.75) compared to migraines without aura (OR = 0.94) and overall migraine risk (OR = 0.92). These effects were consistent among participants who were overweight, did not smoke, and consumed alcohol infrequently, further suggesting that statin effects were not confounding for these established risk factors. Furthermore, they found Lipophilic statins significantly reduced both types of migraines (with a preference for aura) while hydrophilic statins only significantly reduced the risk of migraines without aura. Migraines with aura are linked to decreased cerebral blood flow and cortical depolarization, which trigger inflammation and vessel dilation [34]. Statins, especially lipophilic statins (e.g., lovastatin, simvastatin) inhibit key compounds needed for brain function, such as cholesterol and coenzyme Q, and exhibit anti-inflammatory, antioxidative, and vasomotor regulatory properties [35]. This may explain the lower risk found for migraines with aura compared to those without aura. Add to that the weak ability of hydrophilic statins to cross the blood-brain barrier, lipophilic statins present a better candidate for reducing the risk of migraines with aura more effectively than hydrophilic statins, which is further supported by clinical trials.

The interest in lipid-lowering agents, especially statins, has risen from observing the association between lipids and migraine. In 2021, Liampas et al. conducted a meta-analysis of serum lipids in migraine that found increased total low-density lipoprotein cholesterol (LDL-C), total cholesterol (TC), and triglycerides (TG) [36]. MR studies provided a groundbreaking discovery in research by its ability to infer causal relationships between various drug targets and migraine compared to the correlated nature of observational studies. Hong et al. found no significant association between SNPs related to LDL-C, TC, or TG and migraine. However, they discovered that genotypes of HMGCR related to higher LDL-C levels were associated with migraine risk. This was further elaborated by the findings of Qu et al., based on datasets of the IHGC and FinnGen, who found that HMGCR expression increased migraine risk. Furthermore, they concluded that the association between levels of LDL-C, TC, and TG was only evident through adjustments by HMGCR and not directly linked to these lipids. These findings further support the findings of studies on statin use or HMGCR inhibition and the risk of migraines.

Since the first case report in 2006 of using statins for successful migraine treatment [37], several trials have been conducted to explore the specific efficacy of statins in migraine prophylaxis. Most of the included studies used lipophilic statins (Simvastatin or Atorvastatin) except for Mazdeh et al. who used a hydrophilic statin (Rosuvastatin) for migraine prophylaxis. The preference for lipophilic statins has been explained before. Mazdeh found a significant decrease in several migraine attacks for the combination of propranolol and rosuvastatin compared to propranolol alone. However, this was only a 4-week trial compared to longer trials on lipophilic statins (ranging from 8 to 24 weeks). Statins showed significant effects augmenting migraine medications (propranolol, sodium valproate, and Nortriptyline) in all of the included studies [25, 27, 28], and even more, showed comparable efficacy when used alone in migraine prophylaxis compared to propranolol and sodium valproate [26, 29]. Sherafat et al. conducted the longest trial and found that adding statins to nortriptyline improved not only MMD but also the overall quality of time in 24 weeks of follow-up. Different doses of statins were used in the included studies (ranging between 10 and 40 mg) and future trials are still needed to determine the optimum dose of statins in migraine.

The use of statins showed an overall good safety profile across studies. Hesami et al. found that Atorvastatin had similar prophylaxis efficacy with Sodium Valproate with significantly fewer adverse effects [26]. According to a recent meta-analysis, migraine patients have higher risks of cardiovascular and cerebrovascular events like stroke and myocardial infarction [38]. On the other hand, Statins have shown protective effects against major coronary events and stroke incidence [39, 40]. The potential benefits of statins in preventing migraines for patients with a high risk of cardiovascular events are clinically noteworthy. However, the known adverse events of statins shall be taken into consideration when we consider statins as prophylaxis for migraine including its effects on muscles and increased liver enzymes.

This study had several limitations. First, the number of included trials and patients in them limited the ability to conduct in-depth meta-analysis. Second, the criteria for assessing migraine relief differed among studies. Third, many of the observational studies only included populations of European ancestry, which restricts the generalizability of their findings, except for the Kang et al. study that was based on the Korean population. MR analysis has high statistical power to discover genetic associations, but it is limited by the data from which it gets its conclusions. Fourth, Self-reported data in the Buettner et al. study shall be taken into consideration, while Kang et al. used databases for their nested-case-control study. Finally, the meta-analysis of the efficacy of adjunctive statin use to standard treatment may be limited by including only two studies adding statin to different treatments. However, till the proven scientific use of statins in migraine prophylaxis, exploring the efficacy of adjunctive statins rather than statins as stand-alone treatment may be for the best of the patients.

Future large RCTs are needed to further affirm the efficacy of statins in migraine prophylaxis. Future RCTs shall investigate not only the augmenting effects of add-on statins to traditional migraine drugs but also the effects of statins as stand-alone prophylaxis with migraine, which is promising based on our findings. Long follow-up time and different measurements of efficacy are needed to explore possible long-term adverse events and enable further meta-analysis. Further observational studies on diverse populations are needed to confirm the association between statin use and reduced risk of migraine. MR studies shall take into consideration the use of different datasets from already used datasets in previous studies to avoid overlapping and duplication of findings.

## Conclusion

Statins, already well-established for cardiovascular benefits, emerge as a promising dual-purpose therapy, particularly for patients with overlapping cardiovascular and neurological conditions. This systematic review highlights statins as a promising option for migraine prevention, with observational studies linking HMGCR gene expression to increased migraine risk, suggesting a potential genetic target for future therapies. Randomized controlled trials demonstrate statins’ ability to reduce migraine frequency and severity, offering comparable efficacy to standard treatments with a favorable safety profile. However, the limited number of trials, inconsistent study designs, and varying outcome measures underscore the need for robust evidence. Larger, well-structured RCTs with extended follow-up periods are essential to confirm statins’ efficacy, refine dosing strategies, and establish their role as standalone prophylactic agents.

## Electronic supplementary material

Below is the link to the electronic supplementary material.

Supplementary Material 1

## Data Availability

No datasets were generated or analysed during the current study.

## Abbreviations

NSAIDs: Non-steroidal anti-inflammatory Drugs
HMG‑CoA: 3‑Hydroxy‑3‑Methyl‑Glutaryl Coenzyme A
HMGCR: 3‑Hydroxy‑3‑Methyl‑Glutaryl Coenzyme A reductase
LDL: Low‑Density Lipoprotein
LDL-C: Low-Density Lipoprotein Cholesterol
VLDL: Very low-density lipoproteins
RCT: Randomized Controlled Trials
MR: Mendelian Randomization
PRISMA: Preferred Reporting Items for Systematic Reviews and Meta-Analyses
MeSH: Medical Subject Headings
MMF: Monthly Migraine Frequency
MMD: Monthly Migraine Days
MHD: Monthly Headache Days
ROB: Risk of Bias
NOS: Newcastle-Ottawa Scale
OR: Odds Ratios
CI: Confidence Interval
MD: Mean Difference
IHGC: International Headache Genetics Consortium
TC: Total Cholesterol
TG: Triglycerides

## References

[1] Stovner L, Hagen K, Jensen R, et al (2007) The Global Burden of Headache: A Documentation of Headache Prevalence and Disability Worldwide. Cephalalgia 27:193–210. 10.1111/j.1468-2982.2007.01288.x. 10.1111/j.1468-2982.2007.01288.x 17381554

[2] Lima AM, Sapienza GB, Giraud VO, Fragoso YD (2011) Odors as triggering and worsening factors for migraine in men. Arq Neuropsiquiatr 69:324–327. 10.1590/S0004-282X2011000300011. 10.1590/s0004-282x2011000300011 21625759

[3] Vos T, Abajobir AA, Abate KH, et al (2017) Global, regional, and national incidence, prevalence, and years lived with disability for 328 diseases and injuries for 195 countries, 1990–2016: a systematic analysis for the Global Burden of Disease Study 2016. The Lancet 390:1211–1259. 10.1016/S0140-6736(17)32154-2. 10.1016/S0140-6736(17)32154-2 PMC5605509 28919117

[4] Stewart WF, Linet MS, Celentano DD, et al (1991) Age- and Sex-specific Incidence Rates of Migraine with and without Visual Aura. Am J Epidemiol 134:1111–1120. 10.1093/oxfordjournals.aje.a116014. 10.1093/oxfordjournals.aje.a116014 1746521

[5] Evers S, Áfra J, Frese A, et al (2009) EFNS guideline on the drug treatment of migraine– revised report of an EFNS task force. Eur J Neurol 16:968–981. 10.1111/j.1468-1331.2009.02748.x. 10.1111/j.1468-1331.2009.02748.x 19708964

[6] Tronvik E, Giri S, Young W (2024) Preventive treatment of migraine: Non-specific oral agents. In: Handbook of Clinical Neurology. Elsevier, pp 67–86 10.1016/B978-0-12-823357-3.00009-4 38307673

[7] Vaughan CJ, Gotto AM, Basson CT (2000) The evolving role of statins in the management of atherosclerosis. J Am Coll Cardiol 35:1–10. 10.1016/S0735-1097(99)00525-2. 10.1016/s0735-1097(99)00525-2 10636252

[8] Tousoulis D, Psarros C, Demosthenous M, et al (2014) Innate and Adaptive Inflammation as a Therapeutic Target in Vascular Disease. J Am Coll Cardiol 63:2491–2502. 10.1016/j.jacc.2014.01.054. 10.1016/j.jacc.2014.01.054 24613322

[9] Al-kuraishy HM, Al‐Gareeb AI, Alexiou A, et al (2023) Pros and cons for statins use and risk of Parkinson’s disease: An updated perspective. Pharmacol Res Perspect 11:e01063. 10.1002/prp2.1063. 10.1002/prp2.1063 PMC9944858 36811160

[10] Mady A, Nabil Y, Daoud A, et al (2024) Determining the role of statins in Parkinson’s disease risk reduction and disease modification: A comprehensive meta-analysis of 4 million participants’ data. CNS Neurosci Ther 30:e14888. 10.1111/cns.14888. 10.1111/cns.14888 PMC11298167 39097909

[11] Kursun O, Yemisci M, Van Den Maagdenberg AMJM, Karatas H (2021) Migraine and neuroinflammation: the inflammasome perspective. J Headache Pain 22:55. 10.1186/s10194-021-01271-1. 10.1186/s10194-021-01271-1 PMC8192049 34112082

[12] Jiménez-Jiménez FJ, Alonso-Navarro H, García-Martín E, et al (2024) Oxidative Stress and Migraine. Mol Neurobiol 61:8344–8360. 10.1007/s12035-024-04114-7. 10.1007/s12035-024-04114-7 38499906

[13] Qu K, Li M, Yu P, et al (2024) HMG-CoA reductase is a potential therapeutic target for migraine: a mendelian randomization study. Sci Rep 14:12094. 10.1038/s41598-024-61628-9. 10.1038/s41598-024-61628-9 PMC11130224 38802400

[14] Buettner C, Burstein R (2015) Association of statin use and risk for severe headache or migraine by serum vitamin D status: A cross-sectional population-based study. Cephalalgia 35:757–766. 10.1177/0333102414559733. 10.1177/0333102414559733 25424706

[15] Buettner C, Nir R, Bertisch SM, et al (2015) Simvastatin and vitamin D for migraine prevention: A randomized, controlled trial. Ann Neurol 78:970–981. 10.1002/ana.24534. 10.1002/ana.24534 PMC4715556 26418341

[16] Hong P, Han L, Wan Y (2024) Mendelian randomization study of lipid metabolism characteristics and migraine risk. Eur J Pain 28:978–986. 10.1002/ejp.2235. 10.1002/ejp.2235 38183343

[17] Higgins JPT, Green S, Cochrane Collaboration (2021) Cochrane handbook for systematic reviews of interventions version 6.2 [updated February 2021] Cochrane. Wiley-Blackwell, Chichester, England; Hoboken, NJ

[18] Page MJ, McKenzie JE, Bossuyt PM, et al (2021) The PRISMA 2020 statement: an updated guideline for reporting systematic reviews. BMJ n71. 10.1136/bmj.n71. 10.1136/bmj.n71 PMC8005924 33782057

[19] Ouzzani M, Hammady H, Fedorowicz Z, Elmagarmid A (2016) Rayyan—a web and mobile app for systematic reviews. Syst Rev 5:210. 10.1186/s13643-016-0384-4. 10.1186/s13643-016-0384-4 PMC5139140 27919275

[20] Sterne JAC, Savović J, Page MJ, et al (2019) RoB 2: a revised tool for assessing risk of bias in randomised trials. BMJ l4898. 10.1136/bmj.l4898. 10.1136/bmj.l4898 31462531

[21] Wells, George A, Shea, Beverly, O’Connell D, et al (2004) The Newcastle-Ottawa Scale (NOS) for Assessing the Quality of Nonrandomised Studies in Meta-Analyses.

[22] DerSimonian R, Laird N (1986) Meta-analysis in clinical trials. Control Clin Trials 7:177–188. 10.1016/0197-2456(86)90046-2. 10.1016/0197-2456(86)90046-2 3802833

[23] Higgins JPT, Thompson SG (2002) Quantifying heterogeneity in a meta-analysis. Stat Med 21:1539–1558. 10.1002/sim.1186. 10.1002/sim.1186 12111919

[24] Sterne JAC, Egger M, Smith GD (2001) Systematic reviews in health care: Investigating and dealing with publication and other biases in meta-analysis. BMJ 323:101–105. 10.1136/bmj.323.7304.101. 10.1136/bmj.323.7304.101 PMC1120714 11451790

[25] Ganji R, Majdinasab N, Hesam S, et al (2021) Does atorvastatin have augmentative effects with sodium valproate in prevention of migraine with aura attacks? A triple-blind controlled clinical trial. J Pharm Health Care Sci 7:12. 10.1186/s40780-021-00198-8. 10.1186/s40780-021-00198-8 PMC8015063 33789774

[26] Hesami O, Sistanizad M, Asadollahzade E, et al (2018) Comparing the Effects of Atorvastatin With Sodium Valproate (Divalproex) on Frequency and Intensity of Frequent Migraine Headaches: A Double-blind Randomized Controlled Study. Clin Neuropharmacol 41:94–97. 10.1097/WNF.0000000000000280. 10.1097/WNF.0000000000000280 29746282

[27] Mazdeh M, Mahmudian R, Vafaei SY, et al (2020) Effect of Propranolol with and without Rosuvastatin on Migraine Attacks: A Triple Blind Randomized Clinical Trial. Future Neurol 15:FNL44. 10.2217/fnl-2019-0029.

[28] Sherafat A, Sahebnasagh A, Rahmany R, et al (2022) The preventive effect of the combination of atorvastatin and nortriptyline in migraine-type headache: a randomized, triple-blind, placebo-controlled trial. Neurol Res 44:311–317. 10.1080/01616412.2021.1981105. 10.1080/01616412.2021.1981105 35037597

[29] Medeiros FL, Medeiros PL, Valença MM, Dodick D (2007) Simvastatin for Migraine Prevention. Headache J Head Face Pain 47:855–856. 10.1111/j.1526-4610.2007.00824.x. 10.1111/j.1526-4610.2007.00824.x 17578535

[30] Bi Y, Zhu Y, Tang S, Huang Y (2023) Lipids, lipid-modifying drug target genes and migraine: a Mendelian randomization study. J Headache Pain 24:112. 10.1186/s10194-023-01633-x. 10.1186/s10194-023-01633-x PMC10439594 37596566

[31] Zhang C, He Y, Liu L (2024) Identifying therapeutic target genes for migraine by systematic druggable genome-wide Mendelian randomization. J Headache Pain 25:100. 10.1186/s10194-024-01805-3. 10.1186/s10194-024-01805-3 PMC11167905 38867170

[32] Bjørk MH, Borkenhagen S, Oteiza F, et al (2024) Comparative retention and effectiveness of migraine preventive treatments: A nationwide registry-based cohort study. Eur J Neurol 31:e16062. 10.1111/ene.16062. 10.1111/ene.16062 PMC11235668 37754544

[33] Kang HS, Kim J-H, Kim JH, et al (2024) The Association between Statin Use and Reduced Migraine Likelihood: A Comprehensive Analysis of Migraine Subtypes and Statin Types in a Nationwide Korean Cohort. Pharmaceuticals 17:1056. 10.3390/ph17081056. 10.3390/ph17081056 PMC11357270 39204161

[34] Sanchez Del Rio M, Cutrer FM (2023) Pathophysiology of migraine aura. In: Handbook of Clinical Neurology. Elsevier, pp 71–83 10.1016/B978-0-12-823356-6.00016-0 38043972

[35] Morofuji Y, Nakagawa S, Ujifuku K, et al (2022) Beyond Lipid-Lowering: Effects of Statins on Cardiovascular and Cerebrovascular Diseases and Cancer. Pharm Basel Switz 15:151. 10.3390/ph15020151. 10.3390/ph15020151 PMC8877351 35215263

[36] Liampas I, Mylonas KS, Brotis A, et al (2021) Serum lipid abnormalities in migraine: A meta-analysis of observational studies. Headache J Head Face Pain 61:44–59. 10.1111/head.14039. 10.1111/head.14039 33398889

[37] Liberopoulos EN, Mikhailidis DP (2006) Could Statins Be Useful in the Treatment of Patients With Migraine? Headache J Head Face Pain 46:672–675. 10.1111/j.1526-4610.2006.00293.x. 10.1111/j.1526-4610.2006.00293.x 16643563

[38] Mahmoud AN, Mentias A, Elgendy AY, et al (2018) Migraine and the risk of cardiovascular and cerebrovascular events: a meta-analysis of 16 cohort studies including 1 152 407 subjects. BMJ Open 8:e020498. 10.1136/bmjopen-2017-020498. 10.1136/bmjopen-2017-020498 PMC5875642 29593023

[39] Wang W, Zhang B (2014) Statins for the prevention of stroke: a meta-analysis of randomized controlled trials. PloS One 9:e92388. 10.1371/journal.pone.0092388. 10.1371/journal.pone.0092388 PMC3958535 24643199

[40] Cheung BMY, Lauder IJ, Lau C-P, Kumana CR (2004) Meta-analysis of large randomized controlled trials to evaluate the impact of statins on cardiovascular outcomes. Br J Clin Pharmacol 57:640–651. 10.1111/j.1365-2125.2003.02060.x. 10.1111/j.1365-2125.2003.02060.x PMC1884492 15089818

