# Supplementary material for: Exploring the association between statins use or HMG-CoA reductase inhibition and migraine: a systematic review and meta-analysis
Source: J Headache Pain. 2025 Feb 3;26(1):23. doi: 10.1186/s10194-025-01957-w (PMC11792188; doi:10.1186/s10194-025-01957-w)
Supplement: Supplementary file 1 — Supplementary Material 1 [file 10194_2025_1957_MOESM1_ESM.docx]

**Table1 supplementary: Detailed search strategy**

| Database | Search Terms | N |
| --- | --- | --- |
| PubMed (All fields) | (statin OR Simvastatin OR atorvastatin OR rosuvastatin OR pravastatin OR fluvastatin OR lovastatin OR HMG-CoA OR HMGCR) AND (MIGRAINE) | 70 |
| Scopus (Title, abstract, and keywords.) | (statin OR Simvastatin OR atorvastatin OR rosuvastatin OR pravastatin OR fluvastatin OR lovastatin OR HMG-CoA OR HMGCR) AND (MIGRAINE) | 452 |
| WOS (All fields) | \| (statin OR Simvastatin OR atorvastatin OR rosuvastatin OR pravastatin OR fluvastatin OR lovastatin OR HMG-CoA OR HMGCR) AND (MIGRAINE) \| \| --- \| | 58 |
| Cochrane library (All fields) | (statin OR Simvastatin OR atorvastatin OR rosuvastatin OR pravastatin OR fluvastatin OR lovastatin OR HMG-CoA OR HMGCR) AND (MIGRAINE) | 30 |
